# Improved node culture methods for rapid vegetative propagation of switchgrass (*Panicum virgatum* L.)

**DOI:** 10.1186/s12870-021-02903-z

**Published:** 2021-03-04

**Authors:** Yongqin Wang, Weihong Dong, Malay C. Saha, Michael K. Udvardi, Yun Kang

**Affiliations:** Noble Research Institute, LLC, Ardmore, OK 73401 USA

**Keywords:** Node culture, *In planta* node culture, Micropropagation, Vegetative propagation, Axillary bud, Switchgrass, *Panicum virgatum* L

## Abstract

**Background:**

Switchgrass (*Panicum virgatum* L.) is an important bioenergy and forage crop. The outcrossing nature of switchgrass makes it infeasible to maintain a genotype through sexual propagation. Current asexual propagation protocols in switchgrass have various limitations. An easy and highly-efficient vegetative propagation method is needed to propagate large natural collections of switchgrass genotypes for genome-wide association studies (GWAS).

**Results:**

Micropropagation by node culture was found to be a rapid method for vegetative propagation of switchgrass. Bacterial and fungal contamination during node culture is a major cause for cultural failure. Adding the biocide, Plant Preservative Mixture (PPM, 0.2%), and the fungicide, Benomyl (5 mg/l), in the incubation solution after surface sterilization and in the culture medium significantly decreased bacterial and fungal contamination. In addition, “shoot trimming” before subculture had a positive effect on shoot multiplication for most genotypes tested. Using the optimized node culture procedure, we successfully propagated 330 genotypes from a switchgrass GWAS panel in three separate experiments. Large variations in shoot induction efficiency and shoot growth were observed among genotypes. Separately, we developed an *in planta* node culture method by stimulating the growth of aerial axillary buds into shoots directly on the parent plants, through which rooted plants can be generated within 6 weeks. By circumventing the tissue culture step and avoiding application of exterior hormones, the *in planta* node culture method is labor- and cost-efficient, easy to master, and has a high success rate. Plants generated by the *in planta* node culture method are similar to seedlings and can be used directly for various experiments.

**Conclusions:**

In this study, we optimized a switchgrass node culture protocol by minimizing bacterial and fungal contamination and increasing shoot multiplication. With this improved protocol, we successfully propagated three quarters of the genotypes in a diverse switchgrass GWAS panel. Furthermore, we established a novel and high-throughput *in planta* node culture method. Together, these methods provide better options for researchers to accelerate vegetative propagation of switchgrass.

**Supplementary Information:**

The online version contains supplementary material available at 10.1186/s12870-021-02903-z.

## Background

Switchgrass is a warm-season C4 perennial grass, widely distributed in eastern and central North America [1]. Traditionally used for forage production, restoration of grasslands, and soil conservation, it has also been designated as a bioenergy crop in the United States because of its high biomass production, abiotic stress tolerance and adaptability to marginal lands [1–4]. Being a highly heterozygous and out-crossing allopolyploid species, switchgrass possesses high genotypic and phenotypic variation [5, 6].

Switchgrass is self-incompatible [7], which means that asexual reproduction by vegetative propagation is the only way to maintain a genotype. Vegetative propagation occurs naturally in many plant species [8]. It is widely used by horticulturalists and agricultural companies to rapidly clone plants. For example, in vitro shoot multiplication from nodal explants has been evaluated for rapid propagation of walnut and pecan cultivars with desirable traits [9, 10]. Switchgrass, as well as many other grasses, can produce side shoots or branches, called “tillers”, from the parent shoot. Tillers develop from axillary buds at basal non-elongated internodes [11]. For its simplicity, the most common vegetative propagation method in switchgrass is via tillers. However, when the number of tillers is small and/or there is a need to propagate large quantities of plants, vegetative propagation by either node culture or tissue culture is often performed. Several plant regeneration systems in switchgrass have been established primarily based on the cultivar Alamo [12–16]. Starting materials include mature caryopses, young leaf segments, inflorescences, seedlings, and nodal segments. Recently, a simplified node culture technique for vegetative propagation of switchgrass using a hydroponic system was reported that does not require in vitro tissue culture [17]. Among these methods, the node culture method, with direct shoot production from nodes, has little chance to induce somaclonal variation because it avoids dedifferentiation and re-differentiation that occur in callus culture-based regeneration systems [14]. It is also efficient and appropriate for high-throughput culture of large numbers of genotypes. However, contamination during tissue culture is a challenge due to endophytic bacteria and fungi in explants, which are generally not killed by surface sterilization [18–20].

As part of a large genome-wide association studies, we needed to propagate a diverse set of 436 deeply-sequenced tetraploid switchgrass genotypes. To this end, we developed a modified *ex planta* node culture procedure with reduced contamination and improved efficiency. In addition, a novel high-throughput *in planta* node culture method was established.

## Results

### Effects of antibiotics and fungicides on microbial contamination during node culture

Propagation by dividing tillers is the easiest method for switchgrass propagation. However, obtaining tillers may not be feasible when starting materials are limited and/or large numbers of clonal plants are required. We needed to generate 30–40 copies of each genotype of a switchgrass GWAS panel for both hoop house and greenhouse experiments. We initiated node culture due to lack of tillers and to produce disease-free clonal ramets.

**Fig. 1:**
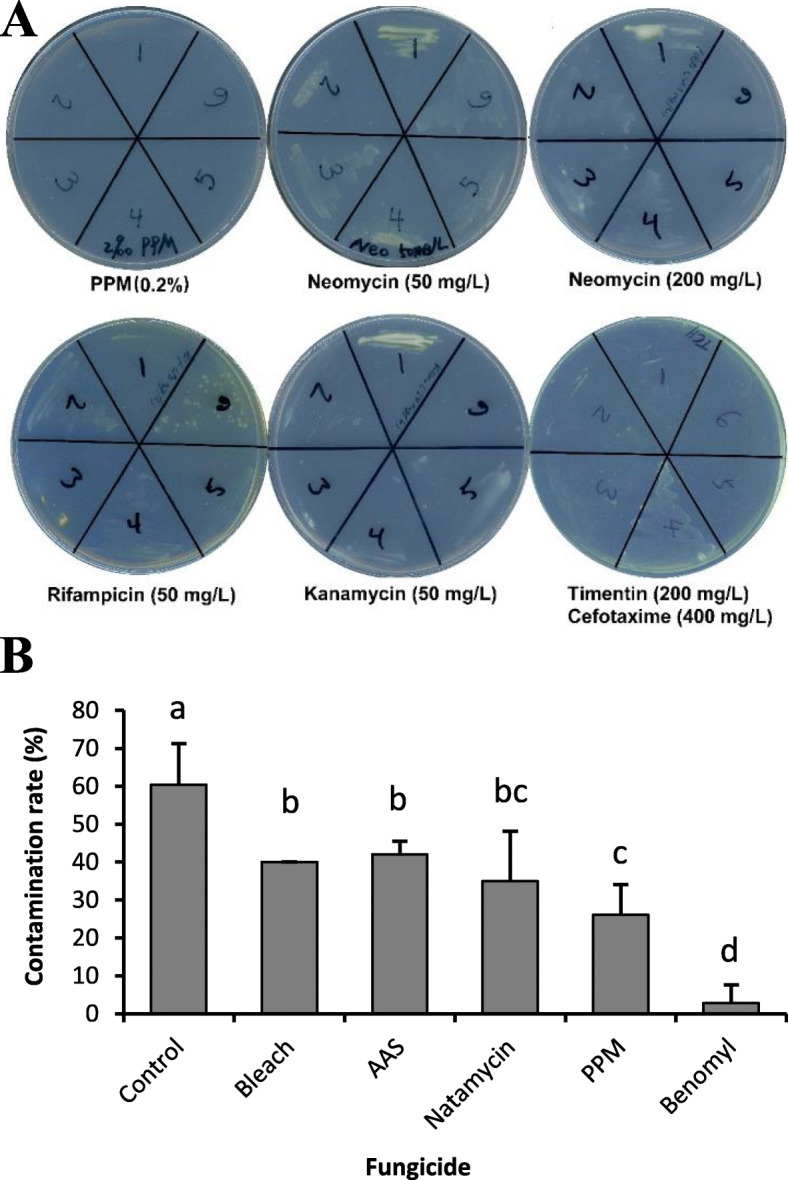
Efficacy comparisons of different antibiotics and fungicides on contamination control. (A) Inhibition of bacterial growth by six different antibiotic treatments. Each plate was inoculated with six different bacterial stains (1–6) that were isolated from contaminated node culture plates. (B) Suppression of fungal contamination by five different fungicides (0.2% bleach, 0.1% AAS, 20 mg/l Natamycin, 0.2% PPM and 5 mg/l Benomyl). Values represent means with standard deviation from three biological replicates, and significant differences are indicated by different letters (*P* < 0.05, ANOVA, LSD). Node samples for each treatments ranged from 24 to 35

In a trial experiment, we attempted to propagate 12 switchgrass genotypes following the published node culture procedure [14], which resulted in severe bacterial and fungal contamination, even though parent plants were healthy and grown under greenhouse conditions. To control bacterial contamination, we tested the inhibitory effects of various antibiotics on six different bacterial colonies that were isolated from contaminated node culture plates (Fig. 1A). Growth of all six strains was completely suppressed in a medium containing 0.2% Plant Preservative Mixture (PPM, Caisson Labs, a robust broad-spectrum biocide), while only one or two strains stopped growth with addition of other antibiotics, including neomycin (50 or 200 mg/l), rifampicin (50 mg/l), kanamycin (50 mg/l), and timentin (200 mg/l) and cefotaxine (400 mg/l) in combination. Notably, 0.2% PPM should be added in the node culture media during the entire process of node culture, because we found absence of PPM or reducing PPM to 0.1% in the shoot subculture medium led to outbreak of bacterial contamination in the later stage. Shoots from most genotypes grew normally at 0.2% PPM, while around 2% of the GWAS genotypes were sensitive to 0.2% PPM, showing yellow leaves or brown leaf tips.

To suppress fungal contamination, five anti-fungal agents were tested by addition to the soaking solution after sterilization as well as to the tissue culture medium. Compared with the non-treatment control, contamination rate was reduced upon all fungicide treatments, and the most dramatic effect was observed with Benomyl (5 mg/l, Sigma) treatment (Fig. 1B). Most fungal contamination was completely suppressed by Benomyl treatment after 2 weeks on culture plates. No negative effect on plant growth was observed with Benomyl (5 mg/l) treatment for all genotypes in the GWAS panel.

### Optimization of the node culture protocol

**Fig. 2:**
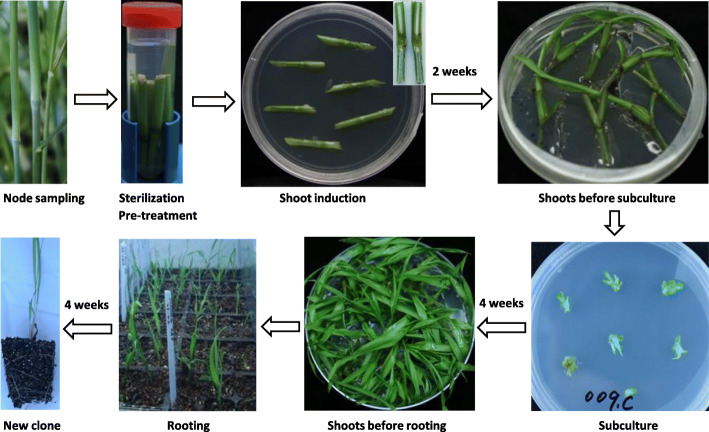
Optimized node culture procedure from Alexandrova et al. [14]. A pretreatment step was added before shoot induction. PPM (0.2%) and Benomyl (5 mg/l) were added to both the pretreatment solution and the node culture medium for contamination control. Shoot-trimming before subculture was recommended for genotypes with fast growing shoots

The node culture protocol was optimized to minimize contamination and increase culture success rate (Fig. 2). First, collecting healthy, undamaged and mature (fully-elongated) node segments from the second elongated node to (but excluding) the top node. In our experience, using nodes very close to the ground or with damaged leaf sheaths increased the contamination rate, while soft and tender young nodes were more likely to be damaged during sterilization, and the top nodes usually produced inflorescences. Second, after 40-min sterilization with bleach, a pre-treatment step of soaking the node materials in sterile water containing 0.2% PPM and 5 mg/l Benomyl was added to reduce bacterial and fungal contamination. Node samples under treatment were kept at 4 °C overnight and up to 5 days. No negative effect on shoot induction was observed with this treatment. Third, for shoot induction, the addition of 0.2% PPM and 5 mg/l Benomyl in the node culture medium and firm contact between the node and the medium were vital for contamination control. In addition, the longer the time on the culture medium, the more bacterial and fungal contamination was observed. Therefore, we avoided keeping node samples on the culture medium for longer than 2 weeks. Fourth, when excising newly-developed shoots for subculture, the basal part of each shoot was left intact, while the shoot was trimmed from top to a final length of 0.5–1.0 cm. By trimming the shoot, more shoots were induced in most genotypes tested (Figure S1). However, for the slow growers, shoots were directly transferred for subculture without trimming. The same node culture medium containing 0.2% PPM and 5 mg/l Benomyl was used for subculture. After culturing for 3–4 weeks, multiple shoots would develop. Repeating the subculture step would produce more shoots. Finally, in the rooting step, no rooting hormone was applied because no significant promoting effect was observed when rooting hormone (Garden Safe TakeRoot Rooting Hormone, Lowe’s) was used on multiple genotypes.

### Propagation of a switchgrass GWAS population by node culture

**Fig. 3:**
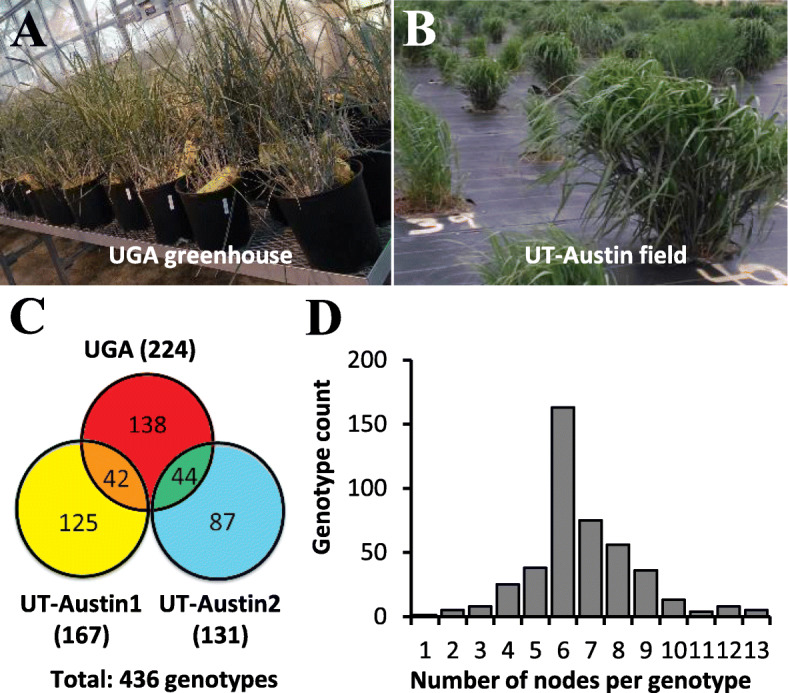
Node sampling of the switchgrass GWAS population. Nodal segments were collected from the UGA greenhouse (**a**) and the UT-Austin field plots (**b**). A total of 436 genotypes were sampled in three sampling events (**c**). For most genotypes, four to ten nodal segments were collected (**d**)

The switchgrass GWAS population, including 436 deeply-sequenced genotypes, were collected and maintained in the field at The University of Texas at Austin (UT-Austin) by the group of Thomas Juenger, while a copy was also grown in a greenhouse at the University of Georgia (UGA). To propagate this population vegetatively, we carried out three node culture experiments (Fig. 3). In the first experiment, nodes from 224 genotypes were sampled from the UGA greenhouse. Shoots were induced from 170 genotypes, of which 149 genotypes had successful root regeneration. Because of no or few plants generated by node culture, 86 genotypes were resampled in the next two experiments. In the second and third sampling experiments, nodes from167 and 131 genotypes were collected from the UT-Austin field plots, respectively. Of these, shoots were induced from 150 and 67 genotypes, and rooted plants were developed from 128 and 64 genotypes, respectively (Table 1). Twenty-nine out of the 42 re-cultured genotypes in the UT-Austin1 experiment (Fig. 3c) developed rooted plants from node culture; and 21 out of the 44 genotypes re-sampled in the UT-Austin2 experiment (Fig. 3c) produced rooted plants. For most genotypes, nodes were collected from one to three tillers. The number of nodes sampled per genotype ranged from one to thirteen, with most genotypes yielding from four to ten (Fig. 3d). In summary (Table 1), 436 genotypes were sampled in total, of which 330 genotypes were successfully propagated by node culture, with a propagation efficiency of 75.7%.

**Table 1:** Summary of three node culture experiment outcomes

Experiment	UGA	UT-Austin1	UT-Austin2
Genotypes sampled	224	167	131
Genotypes produced shoots	170	150	67
Genotypes with rooted plants	149	128	64
Propagation efficiency ^a^	66%	77%	49%

^a^ Propagation efficiency was calculated by dividing the number of genotypes successfully propagated by the total number of genotypes sampled

**Fig. 4:**
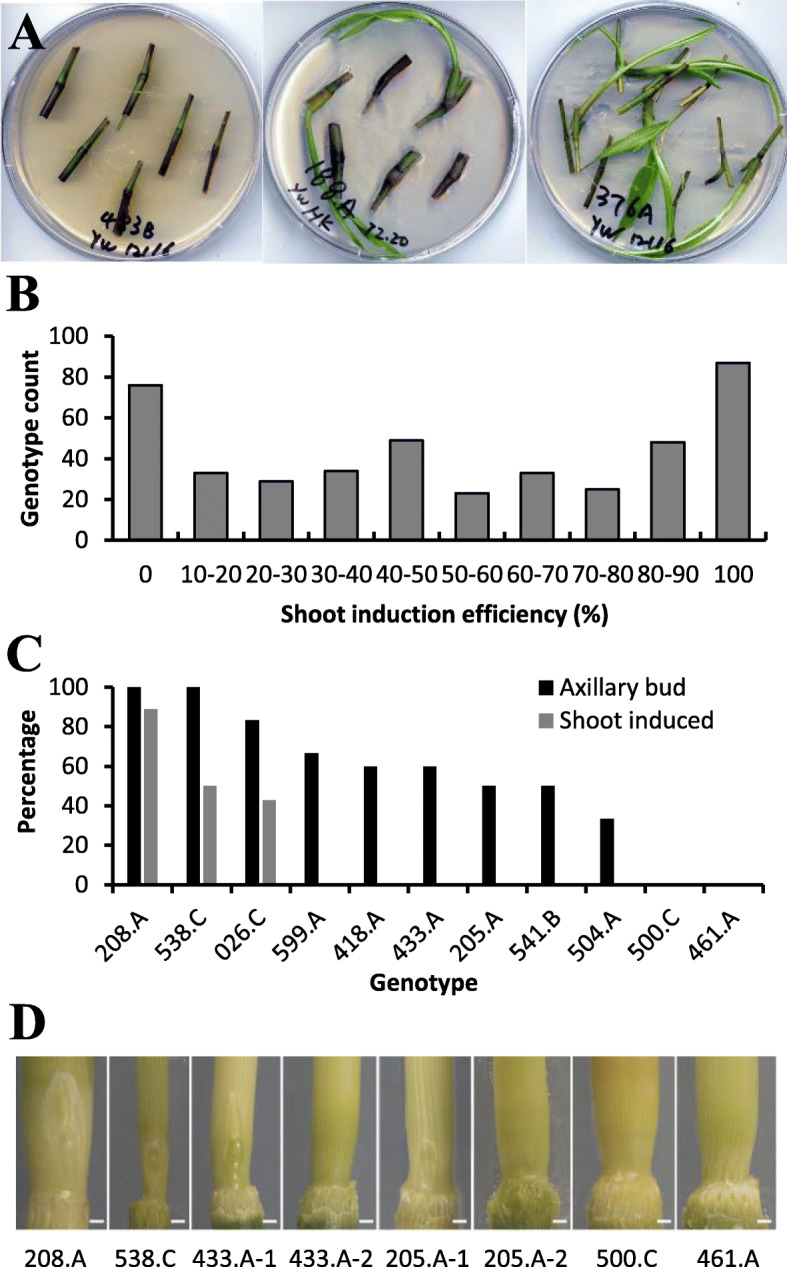
Shoot induction efficiency of the GWAS population. **a** Three representative genotypes showing diverse shoot induction efficiency. **b** Distribution in shoot induction efficiency of the GWAS population. **c** Comparison of the axillary bud rate and shoot induction rate in 11 genotypes. Data were collected from 8 to 18 node samples per genotype. **d** Presence or absence of axillary buds in representative genotypes. Scale bar: 1 mm

Shoot induction efficiency of the GWAS population was analyzed, and large variations were observed among genotypes (Fig. 4a). Though the efficiency spanned from 0 to 100%, the distribution of the number of genotypes was not even (Fig. 4b). Seventy-six genotypes showed zero induction efficiency, while 89 genotypes exhibited 100% efficiency. Among the genotypes that failed in node culture, a small portion (around 10%) was caused by severe bacterial or fungal contamination at the very early shoot induction stage during node culture. However, a number of genotypes did not generate shoots even in the absence of any visible contamination. To understand whether low shoot induction efficiency was correlated with low axillary bud formation rate, we examined axillary buds in eleven genotypes (Fig. 4c and d). For genotype 208.A, all nodes had axillary buds, and 89% of nodes gave shoots. Genotype 538.C had an axillary bud rate of 100%, but only half of the nodes produced shoots during node culture. Similarly, the axillary bud rate for 026.C was twice as high as the shoot induction rate. Six genotypes had axillary bud rates from 33 to 67%, although no shoots were induced in any of them. Two out of the eleven genotypes (500.C and 461.A) had no axillary buds, and no shoots were induced either.

**Fig. 5:**
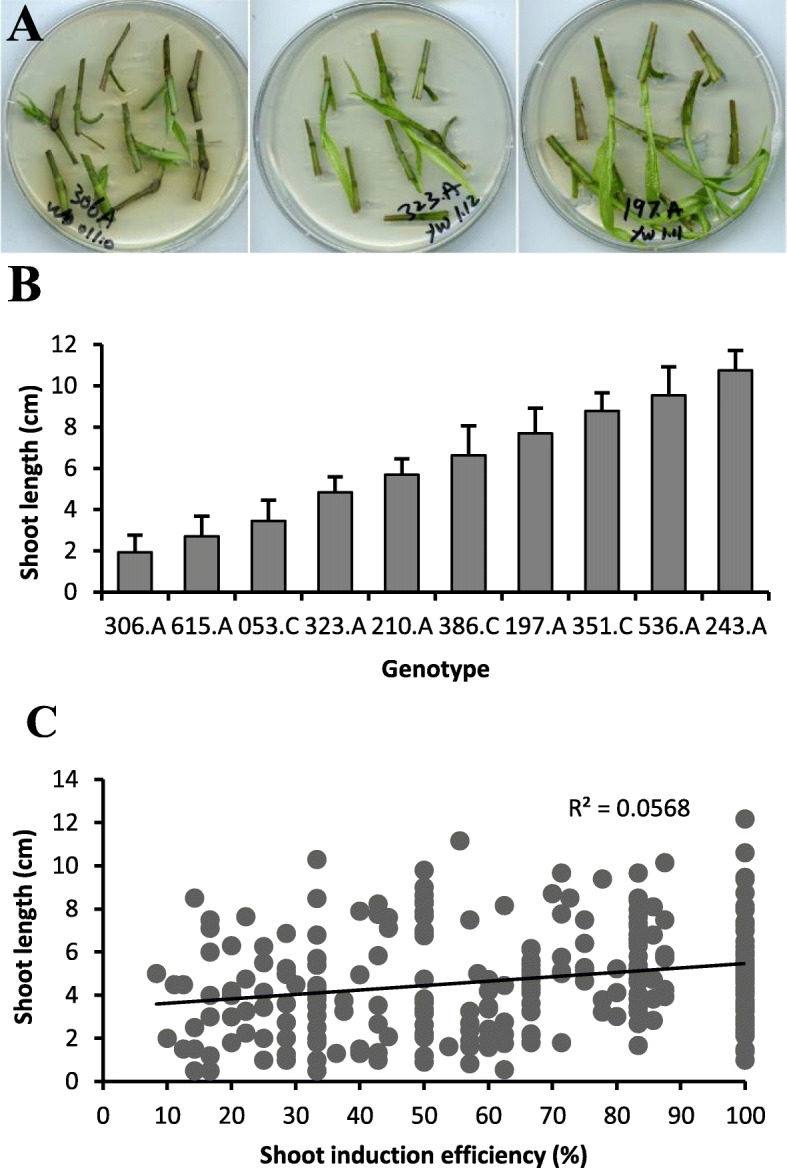
Variation in the shoot growth speed of the GWAS population. **a** Three representative genotypes showing diverse shoot growth speeds. **b** Shoot lengths of 10 representative genotypes after 14 days of culture on the plates. Values are means with standard deviation from 5 to 8 shoots per genotype. **c** Correlation between shoot length and shoot induction efficiency. Data on shoot length and shoot induction efficiency were collected from genotypes with shoots induced after 14 days of culture

Shoots generated during node cultures showed great diversity in growth rate among genotypes (Fig. 5a). After culture for 14 days, shoot length in some genotypes was 2 cm or less, while it was over 10 cm for other genotypes (Fig. 5b). The slow growers remained slow-growing at later stages and had less chance to develop roots, even if they survived the next subculture step. However, no significant correlation was observed between shoot length and shoot induction efficiency (Fig. 5c).

### Development of a novel and high-throughput *in planta* node culture procedure

In addition to optimizing the node culture procedure on plates, we tested a hydroponic node culture system as reported earlier from switchgrass [17], with slight modifications. Sterilized nodal segments of five genotypes were soaked with the bottom half in 1/4 Murashige and Skoog (MS) liquid medium, and no shoots were produced after culturing for up to 4 weeks (Figure S2A). In a parallel experiment using the optimized node culture method as described above, shoots were induced at 100% efficiency from each genotype (Figure S2B). In addition, we tried to directly culture the nodal segments (without sterilization) from six genotypes in a sterilized turface:sand:perlite (2:2:1) mixture. After 2 weeks of culture, a few shoots emerged from one genotype, but they soon stopped growing. Most of the node samples turned yellowish and were contaminated by fungi at the end of culture (Figure S2C). In another experiment, when nodal segments from three genotypes were directly cultured in the soilless media, Metro-Mix 360 (Sun Gro Horticulture), 70% of nodes from 216.A, 80% from 251.C, and 92% from 250.B produced shoots in 2 weeks. After 6 weeks, over half of the shoots from 216.A and 251.C died, and the survivors grew weakly. In contrast, all shoots from 250.B survived, and 80% of them developed roots (Figure S2D). All genotypes tested in the direct node culture experiments above showed 100% of shoot induction efficiency when cultured using the optimized node cultural method.

**Fig. 6:**
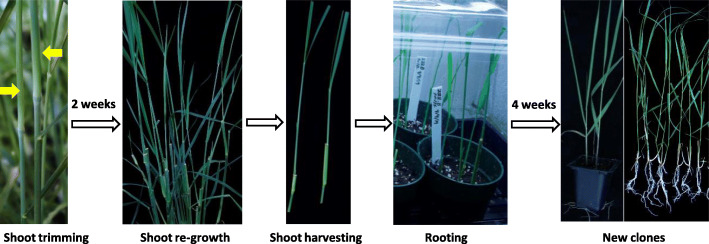
A novel and high-throughput *in planta* node culture method for switchgrass vegetative propagation. This method included three steps, shoot cut back right above the second elongated node (as indicated by the yellow arrows) and allow to regrow, shoot-harvesting, and rooting

In intact switchgrass plants, the basal axillary buds develop into tillers, while the aerial axillary buds from the elongated nodes arrest and remain dormant due to apical dominance. However, we found that if the top part of a tiller was removed, the otherwise dormant axillary bud right below the cutting position would elongate and grow into a new shoot. Taking advantage of this feature, we developed a simple and efficient *in planta* node culture procedure for vegetative propagation of switchgrass (Fig. 6). It includes three simple steps. First, tillers with at least three elongated nodes were selected, and then each tiller was trimmed 3 cm above the second elongated node. New shoots usually developed at the nodes next to the cutting positions on the parent plants within 2 weeks. Second, new shoots with at least one leaf were harvested by cutting 3 cm below the same nodes. Third, new shoots were transferred to soilless media for rooting. Shoots from different genotypes generated by this method exhibited diverse rooting abilities (Figure S3). High rooting efficiency (92–100%) was observed for seven out of ten genotypes within 4 weeks (Table 2). Some genotypes, such as 003.E and 003.C, took 5 weeks or longer to develop roots. The *in planta* node culture method was easy to manipulate, rapid and effective. For its simplicity, we later adopted this method as a routine method for switchgrass propagation and successfully propagated over 100 genotypes.

**Table 2:** Rooting rate of shoots from ten switchgrass genotypes

Genotype	Number of shoots grown	Number of shoots rooted	Rooting rate (%)
160.A	12	11	92
250.B	18	18	100
216.A	7	7	100
250.C	10	10	100
212.A	5	5	100
025.C	9	9	100
614.B	11	11	100
003.E	9	2	22
003.C	16	10	63
419.A	14	11	79

Shoots were produced via the novel *in planta* node culture method. Data were collected at 28 days after transferring shoots into the soilless media

## Discussion

### Pros and cons of different vegetative propagation methods in switchgrass

Vegetative propagation is the only choice to maintain genotypes in out-crossing plant species, such as switchgrass. Each of the several vegetative propagation methods in switchgrass has its advantages and limits. Propagation by tillers is the easiest method to clone switchgrass genotypes, and is the only way to clone genotypes that fail in multiplication by other methods. However, separating tillers causes disturbance and possibly damage to the parent plant. Usually it takes several months for the parent plant to develop a reasonable clump of tillers. In addition, large variations in tillering ability were observed among switchgrass genotypes, which makes it difficult to multiply a complete collection of genotypes. However, micropropagation by node culture is the most efficient method to rapidly clone a switchgrass genotype, with less dependence on and little harm to the parent plant. For example, some genotypes could produce up to seven rooted plant clumps from a single nodal segment within 4 months of culture. New plants produced through this method are much “cleaner” than the parent plant because most pests, pathogens, and endophytes were eliminated during the culture steps. However, some genotypes could not be propagated via this method due to severe fungal or bacterial contamination during node culture, lack of axillary buds, or failure in bud outgrowth. In this study, 106 out of 436 genotypes (24.3%) failed to produce new clones via node culture.

By bypassing the tissue culture step, the *in planta* node culture method is labor- and cost-efficient, free of bacterial and fungal contamination, and shoots produced via it are much more vigorous and have a high (~ 100%) rooting rate. Similar to propagation by tillers, this method depends heavily on the parent plants, and pests and pathogens could be transferred from the parent plants to the new clones. Notably, the clonal plants generated via tillers or *in planta* node culture method could be directly used for experiments, while plants produced through the node tissue culture need to be grown in potting media for a period of time (3 months in our case) to remove the effect of the 6-Benzylaminopurine hormone, which was added during culture, before being used for various experiments.

### Factors affecting node culture efficiency in switchgrass

All the vegetative propagation methods mentioned above rely on the ability of switchgrass to produce axillary buds, which arise from the axillary meristems in the leaf axils [21]. Base on their location on the shoot, axillary buds can be divided into two groups: basal buds, which naturally grow into tillers, and aerial buds, which usually develop a few leaves before entering a dormant state [11, 22]. All node culture methods take advantage of the axillary buds, breaking the dormancy by either applying exogenous cytokinin in the culture medium (such as the tissue culture-based method) or removing the apical dominance (such as the *in planta* method). In addition, mineral nutrients and carbohydrates supplied from the culture medium or through the mother plants promote the growth of the axillary buds. Compared with the tissue culture-based method and the *in planta* node culture method, the lower success rates of various other direct node culture methods (Figure S2) were probably due to the absence of external supply of carbohydrate and hormones. Culturability of a given genotype through various other direct node culture methods can be affected by the internal hormone levels and the storage state of carbohydrates and other nutrients within the nodes themselves, which varies depending on genotypes, growth conditions (field or greenhouse), or developmental stages (young or old). Therefore, various factors should be taken into account when choosing the optimal node culture method.

Large variations in tillering and node culturability were observed in the switchgrass GWAS population. Some genotypes failed to develop axillary buds, and some others failed to develop shoots from axillary buds under the conditions tested. Weaver et al. (2014) found that shoot formation rates varied in Alamo genotypes when cultured hydroponically, and showed a position effect, with highest shoot formation rates (60–100%) observed in nodes at low positions (the second and third basal nodes) in all ten genotypes [17]. Similar node position effect on shoot emergence was also observed during stem propagation of Miscanthus x giganteus [23]. However, using the same cultivar Alamo, Alexandrova et al. (1996) reported that shoots were induced at similar frequency from all nodes, regardless of their position [14]. Although nodes from different positions were cultured together in our study, the position effect was not observed with the 76 and 89 genotypes that showed 0 and 100% shoot induction efficiency, respectively. Our data suggest that axillary buds are necessary but not sufficient for successful shoot regeneration in the node culture. Different switchgrass genotypes had varying abilities to produce axillary buds and to develop shoots from axillary buds, which probably contributed to the large variation in node culturability. All these studies indicate that genotype is a major factor affecting node culture efficiency.

Another factor that significantly affected the culturability was the health status of the mother plants. The striking difference in culturability of the overlapping genotypes between the three node culture experiments (Fig. 3c) was probably directly related to the health status of the mother plants. Many mother plants of the 82 genotypes, newly transferred from the UT-Austin field to the UGA greenhouse, were old and unhealthy when node samples were collected from UGA in early December 2018. In contrast, mother plants of the 42 re-cultured genotypes in the UT-Austin1 experiment were newly-emerged healthy plants when sampled in late April 2019. For the UT-Austin2 experiment, most mother plants were infected by rust when samples were collected in mid July 2019, and as a consequence, a much higher fungal contamination rate was observed in subsequent node culture.

Besides the high fungal contamination rate during culture, another cause for the relatively low culture efficiency in the UT-Austin2 experiment (Table 1) could be genotypes, most of which were upland ecotypes with much thinner stems than the lowland ecotypes. Therefore, our study indicates that culture protocol, genotype, and condition of the mother plants seem to be important factors affecting the success rate of node culture.

Regarding the tissue culture-based vegetative propagation, earlier studies showed growth regulators, media compositions and supplements, hardening mixtures, and culture conditions were important factors influencing the efficiency of the culture system [9, 10, 24–29]. These studies also reported that different cultivars responded differently to the culture treatments, indicating the optimized culture protocols were largely cultivar (or genotype) dependent. In this study, the in vitro node culture procedure was optimized based on results from 12 genotypes, all of which showed around 100% shoot induction rate and rooting rate with our optimized *in vito* node culture procedure (Fig. 2). For shoot induction, we evaluated media containing four combinations of 6-Benzylaminopurine (4.4 μM, 8.9 μM) and Thidiazuron (4.5 μM, 8.9 μM), two combinations of Thidiazuron (4.5 μM, 8.9 μM) and 2,4-Dichlorophenoxyacetic acid (4.5 μM), two sugar types, maltose (20 g/l, 30 g/l), sucrose (20 g/l, 30 g/l), and zero sugar. For rooting, we compared media containing full MS salt and half MS salt, maltose (20 g/l) and sucrose (20 g/l), 6-Benzylaminopurine (0.9 μM), Thidiazuron (0.9 μM), IBA (4.9 μM, 9.8 μM), IAA (5.7 μM, 11.4 μM), NAA (2.7 μM), and zero growth regulator. However, none of these efforts led to further improvement of the current culture protocol. Large-scale in vitro propagation of the GWAS population using this optimized node culture method was successful in 75% of the genotypes. For genotypes that failed due to severe contamination, novel sterilization agents may be tested in future, such as essential oils, silver nanoparticles, thymol and carvacrol [30]. Some switchgrass genotypes failed due to the low shoot growth vigor and low rooting ability. This is contrast to the study in some walnut genotypes, which showed a low shoot growth vigor but high rooting ability during micropropagation [31]. Some genotypes that failed might otherwise be culturable through optimizing the node culture protocol, but it will be time-consuming. For genotypes recalcitrant or genetically incompetent to node culture, other methods should be considered.

### Significance of studying axillary bud and shoot formation in switchgrass

Flexibility in plant development and architecture is part of the adaptation strategy for plants to adjust to the prevailing environmental conditions. Plant shoot architecture is largely determined by the number and activity of axillary meristems and the growth characteristics of the branches that develop from axillary buds [21]. Shoot branching traits directly affect the shoot architecture and biomass yield. In switchgrass, tillers contribute greatly to the shoot biomass, while aerial branches rarely develop in intact plants, but might be an important biomass contributor at certain conditions, such as after the top part of the shoots are grazed or harvested. Plant branching is genetically controlled by a complex regulatory network involving phytohormones and transcription factors [21, 22, 32]. Many studies showed that auxins, cytokinins and strigolactones are central to the control of axillary bud activation [22, 32]. In strawberry runner, high levels of auxin maintain the dormancy of the axillary buds, while cytokinin breaks dormancy and promotes the axillary meristem [33]. In switchgrass, evidence suggested that the *miR156-SPL4* module predominantly regulates aerial axillary bud formation and controls shoot architecture [11]. Overexpression of miR156 or down-regulation of *SPL4* by stable transformation in switchgrass promoted aerial bud formation and changed shoot architecture [11]. With rich variation in tillering ability, axillary bud formation, and response to shoot induction during node culture, the switchgrass GWAS population is an excellent resource to investigate mechanisms and genes controlling axillary bud and shoot formation.

## Conclusion

In this study, to accelerate vegetative propagation of a switchgrass GWAS panel, a micropropagation method via node culture was optimized to minimize microbial contamination and increase propagation efficiency. Treatment of nodal segments with 0.2% PPM and 5 mg/l Benomyl after surface sterilization and during culture significantly decreased bacterial and fungal contamination and increased the success rate of culture. Shoot-trimming before sub-culture promoted shoot multiplication in most genotypes. Using the optimized node culture method, 330 out of 436 genotypes in a switchgrass GWAS panel were successfully cloned, with a success rate of 75.7%. In addition, we developed an easy and high-throughput *in planta* node culture method by inducing shoot formation from aerial axillary buds on the parent plant before rooting, which skipped the tissue culture step and only included three simple manipulations: node-trimming, shoot-harvesting, and rooting. According to our knowledge, the *in planta* node culture method developed in this study is novel. In addition, this method has great potential to be applied to other plant species, since apical donimance is widely observed in different plant species and the underlying mechanism controlling bud dormancy is generally common [22, 32]. Considering the advantages and disadvantages of each vegetative propagation method, our study not only provides more vegetative cloning tools to switchgrass researchers, but also potentially benefits researchers in vegetative propagation of other plant species. We demonstrated for the first time that micropropagation of a large and diverse natural collections of switchgrass via node culture is feasible.

## Methods

### Plant materials and node sample collection

All the accessions of the switchgrass GWAS population used in this study were originally provided by Dr. Thomas Juenger from UT-Austin [34]. To propagate the GWAS population, we performed three node culture experiments. In the first experiment, nodal segments were collected in early December of 2018 from a greenhouse of UGA. Node samplings for the second and third experiments were conducted in late April and mid July 2019, respectively, and both were from the field of UT-Austin. After sampling, node samples were immediately stored in 50 ml Falcon Conical Centrifuge Tubes, and kept at 4 °C before use.

### Test of antibiotics and fungicides for contamination control

Six different endophytic bacterial strains were selected from contaminated node culture explants based on the color and growth speed. Bacteria were streaked on node culture medium (Table S1) supplemented with various antibiotics at mentioned concentrations, including neomycin (50 or 200 mg/l, PhytoTech Labs), rifampicin (50 mg/l, PhytoTech Labs), kanamycin (50 mg/l, PhytoTech Labs), timentin (200 mg/l, PhytoTech Labs) and cefotaxine (400 mg/l, PhytoTech Labs), and 0.2% PPM (Plant Preservative Mixture, Caisson Labs), and incubated at 28 °C for 5 days. For the anti-fungal test, after sterilization node segments were soaked overnight in sterile water (as control), or sterile water containing 0.2% bleach, 0.1% AAS (Antibiotic Antimycotic Solution, Sigma), 10 mg/l natamycin (PhytoTech Labs), 0.2% PPM, or 5 mg/l Benomyl (Methyl 1-(butylcarbamoyl)-2-benzimidazolecarbamate, Sigma). Next, treated nodal explants were cultured on node culture medium containing the same fungicide at the same concentration. Fungus contamination was counted after culturing for 2 weeks. ANOVA and LSD tests were performed in Microsoft Excel to determine the statistically significant difference among fungicide treatments.

### Node culture protocol

The node culture protocol in this study was modified based on the publication of Alexandrova et al. [14]. Healthy and clean nodes were excised 3 cm above and 3 cm below each culm node, then surface-sterilized with 30% (v:v) commercial bleach (containing 0.1% Tween 20) by shaking at 100 rpm and room temperature for 40 min, followed by rinsing twice with sterile water. The node samples were then soaked in sterile water containing 0.2% PPM (v:v) and 5 mg/l Benomyl at 4 °C overnight or up to 5 days. Next, each nodal segment was trimmed off 1 cm from each end, split longitudinally into halves with a scalpel, and then transferred to the node culture medium (Table S1) containing 0.2% PPM (v:v) and 5 mg/l Benomyl, with the cut surface facing down and firmly touching but not completely emerged in the medium. The split nodes were cultured for 2 weeks at 24 °C under 16 h photoperiod and 200 μmol m^− 2^ s^− 1^ light intensity. In the next step, newly developed shoots were separated and transferred onto the same culture medium for multiplication. Multiple shoots would be ready for rooting or subculture in 3–4 weeks. For rooting, shoots were grown in the potting medium Metro-Mix 360 and kept in a greenhouse at 26 °C under 16 h photoperiod, 300–500 μmol m^− 2^ s^− 1^ light intensity, and 80% humidity condition. High humidity condition was applied by mist-spraying for 8 s every 30 min with an automatic sprinkler system, or by covering with a dome and watering regularly. After rooting, plants were transferred to another greenhouse for regular growth at 26 °C / 22 °C day and night temperature, 14 h photoperiod, and 300–500 μmol m^− 2^ s^− 1^ light intensity.

### *In planta* node culture protocol

First, tillers with at least three elongated nodes were selected and cut back 3 cm above the second elongated node. Second, after 2 weeks of growth, new shoots with one or two leaves, which emerged from the node right below the cutting position, were harvested 3 cm below the nodes. To reduce transpiration, the leaf blades were partially trimmed if they were too large. Finally, shoots were transferred to Metro-Mix 360 for rooting under the above described high humidity conditions.

### Other modified direct node culture methods

Five genotypes from the switchgrass GWAS population were tested in a modified hydroponic node culture experiment. For each genotype, 20 nodal segments were sampled from plants that were grown in the greenhouse at Noble Research Institute, LLC. After sterilization and pre-treatment, half of the node samples from each genotype were cultured on plates following the “Node culture protocol”, and half were cultured in 50 ml Falcon Conical Centrifuge Tubes, with the bottom half soaked in 5 ml 1/4 MS liquid medium. Node samples from both treatments were cultured at 24 °C under 16 h photoperiod and 200 μmol m^− 2^ s^− 1^ light intensity. In another test experiment, 15–20 nodal segments (3 cm below and 3 cm above each node) were collected from six switchgrass genotypes each, and were directly cultured in a sterilized turface:sand:perlite (2:2:1) mixture, with each node completely buried (2 cm deep) in the medium. Similarly, 10–20 node samples from three switchgrass genotypes were directly cultured in Metro-Mix 360. All the newly-grown nodes were kept in greenhouse under the same high humidity conditions as described above for rooting.

### Data collection and analysis

Visible shoots were counted and shoot length was measured after 14 days of culture. Shoot induction efficiency of a switchgrass genotype was calculated by dividing the number of shoots induced by the number of nodes cultured. If two or more shoots were generated from the two halves of a single node, they were counted as one. To analyze the effect of shoot trimming on shoot multiplication, shoots were trimmed from the top to 0.5–1 cm in length before being transferred for subculture. The number of shoots were counted after culturing for four more weeks. Student’s t-test determined the statistically significant difference between the two treatments (trim and no-trim). For axillary bud examination, the sheath was carefully removed from the node segment by hand, and axillary bud was observed and photographed under a Leica S6D Stereo Microscope.

## Supplementary Information

**Additional file 1: Figure S1.** Effect of shoot trimming on shoot multiplication. By trimming the shoot before subculture, more shoots were induced in three out of the four genotypes tested. Significance (*p ≤ 0.05, **p ≤ 0.01) was analyzed by Student’s test with sample size ranging from 5 to 7.

**Additional file 2: Figure S2.** Other node culture methods tested. (A) Switchgrass nodes from five genotypes were cultured for two weeks by a hydroponic node culture method modified from Weaver et al. [17]. (B) Shoots were induced at 100% efficiency from the same five genotypes as shown in (A) in two weeks of culture by the optimized node culture method. (C) Nodes from six genotypes were cultured in a turface:sand:perlite (2:2:1) mix for three weeks. (D) Nodes from three genotypes were cultured in Metro-Mix 360 for six weeks.

**Additional file 3: Figure S3.** Genotypes showing diverse rooting speed. (A-D) Shoots from four genotypes, generated by the novel *in planta* node culture method, were rooted for 28 days.

**Additional file 4: Table S1.**

## Data Availability

All data generated or analyzed during this study are included in this published article. The datasets used and/or analyzed during the current study are available from the corresponding author on reasonable request.

## Abbreviations

GWAS: Genome-wide association studies
PPM: Plant Preservative Mixture
UT-Austin: The University of Texas at Austin
UGA: University of Georgia
MS: Murashige and Skoog
AAS: Antibiotic Antimycotic Solution
CBI: Center for Bioenergy Innovation
DOE: Department of Energy
OBER: Office of Biological and Environmental Research
