# Supplementary material for: Improved node culture methods for rapid vegetative propagation of switchgrass (Panicum virgatum L.)
Source: BMC Plant Biol. 2021 Mar 4;21:128. doi: 10.1186/s12870-021-02903-z (PMC7931530; doi:10.1186/s12870-021-02903-z)
Supplement: Supplementary file 1 — Additional file 1: Figure S1. Effect of shoot trimming on shoot multiplication. By trimming the shoot before subculture, more shoots were induced in three out of the four genotypes tested. Significance (*p ≤ 0.05, **p ≤ 0.01) was analyzed by Student’s test with sample size ranging from 5 to 7. [file 12870_2021_2903_MOESM1_ESM.pptx]

## Slide 1
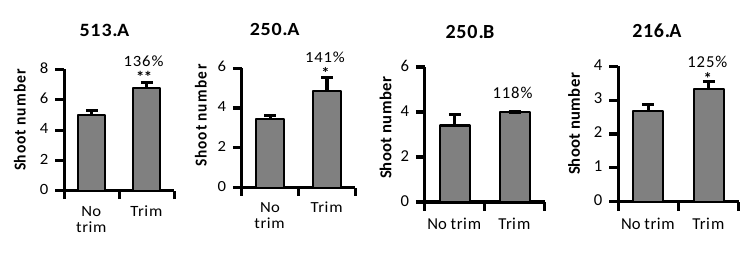

### Chart: 513.A
| Category | |
|---|---|
| No trim | 5.0 |
| Trim | 6.8 |
### Chart: 250.A
| Category | |
|---|---|
| No trim | 3.4285714285714284 |
| Trim | 4.833333333333333 |
### Chart: 216.A
| Category | |
|---|---|
| No trim | 2.6666666666666665 |
| Trim | 3.3333333333333335 |
### Chart: 250.B
| Category | |
|---|---|
| No trim | 3.4 |
| Trim | 4.0 |
