# Supplementary figures and images for: Improved node culture methods for rapid vegetative propagation of switchgrass (Panicum virgatum L.)
Source: BMC Plant Biol. 2021 Mar 4;21:128. doi: 10.1186/s12870-021-02903-z (PMC7931530; doi:10.1186/s12870-021-02903-z)

## Slide 1
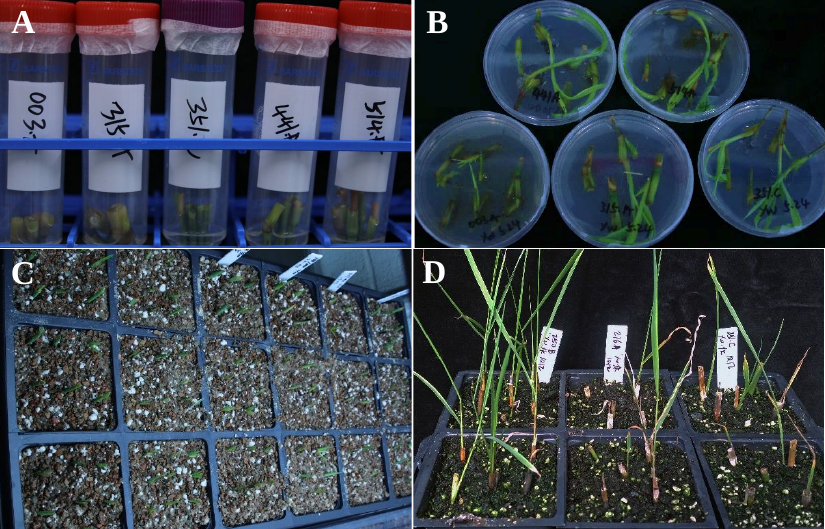

B
A
D
C

Supplement: Supplementary file 2 — Additional file 2: Figure S2. Other node culture methods tested. (A) Switchgrass nodes from five genotypes were cultured for two weeks by a hydroponic node culture method modified from Weaver et al. [17]. (B) Shoots were induced at 100% efficiency from the same five genotypes as shown in (A) in two weeks of culture by the optimized node culture method. (C) Nodes from six genotypes were cultured in a turface:sand:perlite (2:2:1) mix for three weeks. (D) Nodes from three genotypes were cultured in Metro-Mix 360 for six weeks. [file 12870_2021_2903_MOESM2_ESM.pptx]

## Slide 1
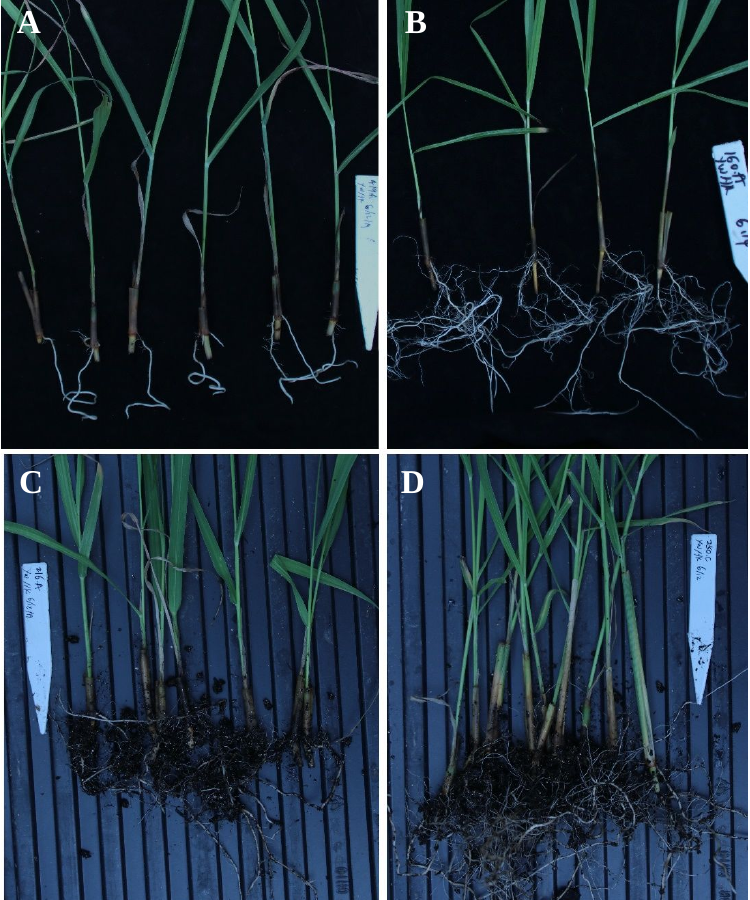

A
B
C
D

Supplement: Supplementary file 3 — Additional file 3: Figure S3. Genotypes showing diverse rooting speed. (A-D) Shoots from four genotypes, generated by the novel in planta node culture method, were rooted for 28 days. [file 12870_2021_2903_MOESM3_ESM.pptx]
