# Supplementary material for: Improved node culture methods for rapid vegetative propagation of switchgrass (Panicum virgatum L.)
Source: BMC Plant Biol. 2021 Mar 4;21:128. doi: 10.1186/s12870-021-02903-z (PMC7931530; doi:10.1186/s12870-021-02903-z)
Supplement: Supplementary file 4 — Additional file 4: Table S1. [file 12870_2021_2903_MOESM4_ESM.docx]

**Table S1** Components of the node culture medium for switchgrass shoot induction and subculture.

| **Medium component** | **Concentration** |
| --- | --- |
| **MS Basal medium with vitamins** (PhytoTech Labs) | 4.43 g/l |
| **D-Maltose** (PhytoTech Labs) | 30 g/l |
| **MES** (Sigma) | 0.5 g/l |
| **6-Benzylaminopurine** (PhytoTech Labs) | 12.5 uM |
| **Micropropagation Agar-Type I** (Caisson Labs) | 8 g/l |
| **pH** | 5.7 |
